# Prognostic signature of lung adenocarcinoma based on stem cell-related genes

**DOI:** 10.1038/s41598-020-80453-4

**Published:** 2021-01-18

**Authors:** Zhanghao Huang, Muqi Shi, Hao Zhou, Jinjie Wang, Hai-Jian Zhang, Jia -Hai Shi

**Affiliations:** 1 Nantong Key Laboratory of Translational Medicine in Cardiothoracic Diseases, and Research Institution of Translational Medicine in Cardiothoracic Diseases, Affiliated Hospital of Nantong University, Nantong, 226001 Jaingsu China; 2 Department of Thoracic Surgery, Affiliated Hospital of Nantong University, Nantong, 226001 Jiangsu China; 3 Research Center of Clinical Medicine, Affiliated Hospital of Nantong University, Nantong, 226001 Jiangsu China; 4 Medical College of Nantong University, Nantong, 226001 Jiangsu China

**Keywords:** Cancer, Immunology, Stem cells

## Abstract

Lung adenocarcinoma (LUAD) is characterized by high infiltration and rapid growth. The function of the stem cell population is to control and maintain cell regeneration. Therefore, it is necessary to study the prognostic value of stem cell-related genes in LUAD. Signature genes were screened out from 166 stem cell-related genes according to the least absolute shrinkage operator (LASSO) and subsequently multivariate Cox regression analysis, and then established risk model. Immune infiltration and nomogram model were used to evaluate the clinical efficacy of signature. A signature consisting of 10 genes was used to dichotomize the LUAD patients into two groups (cutoff, 1.314), and then validated in GSE20319 and GSE42127. There was a significant correlation between signature and clinical characteristics. Patients with high-risk had a shorter overall survival. Furthermore, significant differences were found in multiple immune cells between the high-risk group and low-risk group. A high correlation was also reflected between signature and immune infiltration. What’s more, the signature could effectively predict the efficacy of chemotherapy in patients with LUAD, and a nomogram based on signature might accurately predict the prognosis of patients with LUAD. The signature-based of stem cell-related genes might be contributed to predicting prognosis of patients with LUAD.

## Background

Lung adenocarcinoma refers to a malignant tumor originating from lung epithelial tissue, which is a type of non-small cell lung cancer. In recent years, the incidence rate has gradually increased. In addition, due to the limitations of diagnosis and treatment, the mortality rate of LUAD ranks first in malignant tumors^1^. Tumor stem cells refer to cells that have self-renewal ability and can produce heterogeneous tumor cells, which play a significant role in tumor survival, proliferation, metastasis, and recurrence^2,3^. The ability of tumor stem cells to move and migrate makes tumor cells migration possible, at the same time, cancer stem cells can stay dormant for a long time and have a variety of drug-resistant molecules, but are not sensitive to external physical and chemical factors that kill tumor cells, which leads to the result that tumors often relapse after conventional cancer treatment eliminates most common tumor cells^4,5^. So, genes related to stem cells should also have these characteristics.

The treatment plan and survival period of patients with LUAD are affected by many factors, but the TNM stage of tissue cells may be one of the vital factors in determining the treatment plan and estimating prognosis. TNM stage is based on anatomy and is a description of the cumulative range of tumors. However, it should be emphasized that the TNM stage also has shortcomings including the uneven source of case data and the relatively complicated stage of N. With the gradual development of diagnosis and treatment technology, we found that molecular markers have a greater prognosis for patients. Studying the genetic functions and pathways of LUAD could contribute to establishing prognostic markers and therapeutic targets, which could accurately and comprehensively predict the prognosis of LUAD^6^. Therefore, the idea, which constructed signature through cancer stem cell-related genes provides a new direction for the diagnosis and treatment of LUAD and the regulatory mechanism of stem cell-related genes still requires further digging.

In this research, we constructed a signature of 10 genes as a prognostic target for lung adenocarcinoma. Meanwhile, we analyzed the types of immune cells in LUAD, given that multiple pathways in the gene enrichment analysis are related to immunity, to understand the connection between stem cell-related genes and the immune microenvironment.

## Materials and methods

### Data acquisition and selection

The RNA-sequencing and clinical traits information of LUAD were obtained from The Cancer Genome Atlas (TCGA) database (https://portal.gdc.cancer.gov) and Gene Expression Omnibus (GEO) database (https://www.ncbi.nlm.gov/geo/) that were served as training cohort and validation cohort, respectively. Both data sets were whole-genome sequencing, and the sequenced data included tumor cells and non-tumor cells. This study mainly focused on cancer stem cells rather than normal stem cells. Cancer stem cells are cells in tumors that have the ability to self-renew and produce heterogeneous tumors and are part of tumor cells. Therefore, the sequencing data of both data sets contained the data of cancer stem cells. The FPKM (Fragment Per Kilobase per Million) data with level 3 from the TCGA database was used in this study. After classification and regularization, there were 497 tumor samples in the TCGA database. At the same time, when merging clinical information, missing and incomplete samples were deleted. Besides, 166 tumor stem cell-related genes were downloaded from the cancerSEA database^7^ to prepare for further signature construction. GSE30219 was conducted by GPL570 (Affymetrix Human Genome U133 Plus 2.0 Array)^8^. GSE42127 was conducted by GPL6884 (Illumina HumanWG-6 v3.0 expression bead chip)^9^.

### Signature construction and verification

It was worth emphasizing that the RStudio was an indispensable key tool for us to construct and verify a signature next. The signature was established by a two-step method, the first step was least absolute shrinkage operator (LASSO) Cox regression, using the “glmnet” package (versions 3.0.1), and the second step was multivariate Cox regression, using the “survival” package (versions 3.1.8). Patients were divided into low-risk and high-risk groups based on the cutoff of risk score, which was calculated by formula as follows: HR 1 × gene 1 expression + HR 2 × gene 2 expression … + HR n × gene n expression^10^. In the TCGA and GEO cohorts, the risk curve was drawn to describe further the relationship between the patients' risk value and survival states and protein expression, the Kaplan–Meier curve and ROC curve were used to verify the reliability of the signature^11^.

### Gene set enrichment analysis (GSEA)

GSEA is a method used to evaluate the distribution trend of genes in the gene list sorted by phenotype correlation and to understand gene positioning, function, and biological significance. The GSEA (https://www.gsea-msigdb.org/gsea/downloads.jsp) analysis method used a predefined gene set, usually from functional annotations or the results of previous experiments, to rank the genes according to the degree of differential expression in the two types of samples, and then checked whether the preset gene set was at the top of the ranking list or bottom enrichment. We presented the GO term and the KEGG pathway of the signature which was constructed by stem cell-related genes to further analyze its possible biological functions^12^. The number of permutations was set to 1000, and our selection criteria are closely related to a nominal P-value (p < 0.05).

### Immune infiltration analysis

TIMER database, providing six types of immune cell infiltration and using RNA-Seq expression profiling data to detect immune cell infiltration in tumor tissue, was used to appraise potential links between risk grouping and tumor-infiltrating immune cells (TIICs). Deconvolution is a newly released statistical method that allows TIMER to infer the incidence of TICC from gene expression profiles. CIBERSORT (http://cistrome.shinyappes), a deconvolution algorithm, can estimate the cell composition of complex tissues based on standardized gene expression data, and the method can be used to analyze specific cell types. With CIBERSORT, we can visualize the composition of immune cells in tumor samples of LUAD, and standard annotation files established gene expression datasets. P-value (p < 0.05) was a significant criterion to determine the type of immune cells affected by grouping^13^.

### Analysis of therapeutic efficacy and mRNA expression-based stemness index (mRNAsi)

Some patients from TCGA recorded the results of the evaluation of the efficacy after the first treatment of radiotherapy and chemotherapy, which also provided a direction for us to verify the reliability of the signature in terms of efficacy. According to Response Evaluation Criteria in Solid Tumors (RECIST) and risk score, this part of patients was classified to compare whether there were differences between different therapeutic effects^14^. In recent years, literature has proposed the concept of mRNA expression-based stemness index (mRNAsi), which was calculated by a predictive model with an OCLR algorithm based on pluripotent stem cell samples from the Progenitor Cell Biology Consortium dataset (https://bioinformaticfmrp.github.io/PanCanStem_Web/). Specifically, the Spearman correlation algorithm (RNA expression data) contributed to the stem index model to score LUAD samples in the TCGA dataset. The stem indices were then mapped to the [0, 1] range by using a linear transformation that subtracted the minimum and divide by the maximum. The index is closer to 1, which indicated that the cell differentiation was worse, and the characteristic of stem-cell related genes was stronger. We merged mRNAsi into our signature to compare whether there was a difference between low- and high- risk groups^15^.

### Clinical correlation analysis

Univariate and multivariate Cox regression analyses were used to determine independent predictors of OS in LUAD. The predictive value of signature and other clinical factors were evaluated by the area under the ROC curve. Besides, we have developed a nomogram containing risk scores and clinical information to transform the prognostic value of the signature into clinical use. The nomogram was internally validated using bootstraps with 1000 resamples. The nomogram was composed of independent prognostic factors that were previously screened out, using the “rms” package (version 5.1.4). Each factor was assigned a weight according to its influence on the prognosis. According to the weight of each factor, the corresponding score was obtained to predict the patient’s 1, 3, 5-year survival rate. The higher the score, the worse the prognosis.

## Result

### Construction of Signature in TCGA

**Figure 1:**
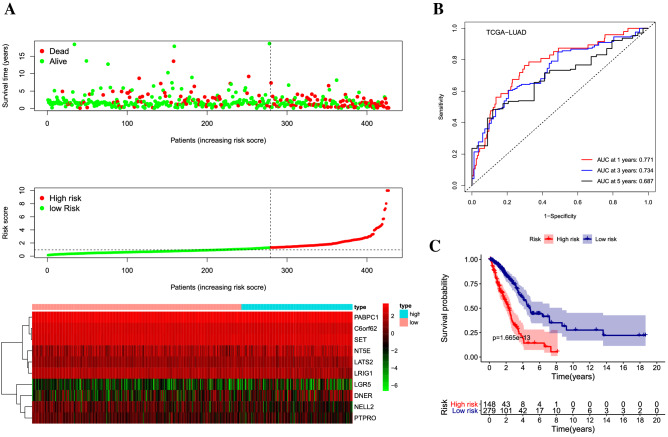
Construction of signature. (**A**) The risk curve in the TCGA cohort displayed the patients’ risk score, survival time, and status, and expression of stem cell-related genes. The scales represented the expression level of each gene in each sample, which was established based on z-score transformed expression data. (**B**) ROC curve illustrated the risk prediction of the signature for 1, 3, and 5-year in the TCGA cohort. (**C**) Kaplan–Meier survival revealed the overall survival among different risk stratification groups.

All cancer stem cell-related genes were downloaded from CancerSEA (http://biocc.hrbmu.edu.cn/CancerSEA/home.jsp). Nineteen stem cell-related genes associated with OS (p < 0.05) were measured as predictive stem cell-related genes for LASSO analysis (Supplementary Information 1–2). Through multivariate COX regression, we select ten stem cell-related genes to construct a robust signature for LUAD (Tables 1, 2). The calculation formula of the risk score is as follows: risk score = 0.578 × expression C6orf62 + 1.24 × expression DNER + 0.737 × expression NELL2 + 1.404 × expression LATS2 + 1.202 × expression LGR5 + 0.676 × expression PTPRO + 0.718 × expression LRIG1 + 1.306 × expression PABPC1 + 1.126 × expression NT5E + 1.458 × expression SET. In the calculation, the mRNA expression value (FPKM) was used to calculate the risk score. According to the cutoff (1.314) of risk scores, patients in TCGA were divided into low-risk group and high-risk group^16^. The risk curve can clearly show the relationship between survival status, survival time, and expression of stem cell-related genes and risk score^17^ (Fig. 1A). The area under the ROC curve for 1, 3, 5-year were 0.771, 0.734, 0.687 (Fig. 1B). In ROC analysis, the survival status of the same patients at 1, 3, and 5 years is inconsistent, which also leads to their inconsistency in AUC at one, three, and five years. In fact, AUC may not necessarily decline with increasing time, but may also increase. Herein, our study shows that AUC decreases gradually with increasing time. The survival analysis suggested that the overall survival rate of the low-risk group was higher than that of the high-risk group (P < 0.001). The 5-year survival rate of the low-risk group was close to 50%, while the 5-year survival rate of the high-risk group was only 20% (Fig. 1C).

**Table 1:** ENSEMBL/Entrez gene ID.

ID	EntrezID
C6orf62	81,688
DNER	92,737
NELL2	4753
LATS2	26,524
LGR5	8549
PTPRO	5800
LRIG1	26,018
PABPC1	26,986
NT5E	4907
SET	6418

**Table 2:** Independent factors in the signature.

ID	coef	HR	HR.95L	HR.95H	P value
C6orf62	− 0.73221	0.480847	0.312891	0.738959	0.000838
DNER	0.195561	1.215993	1.073065	1.377959	0.002174
NELL2	− 0.34583	0.707635	0.533484	0.938635	0.016425
LATS2	0.38125	1.464113	1.100657	1.947588	0.008826
LGR5	0.251941	1.28652	1.076618	1.537345	0.005566
PTPRO	− 0.44697	0.639564	0.430824	0.94944	0.026599
LRIG1	− 0.26479	0.767366	0.633265	0.929864	0.006893
PABPC1	0.246554	1.279608	0.983828	1.664311	0.066004
NT5E	0.132706	1.141914	1.006795	1.295166	0.038888
SET	0.414622	1.513798	1.03377	2.216727	0.033119

### Validation of the signature in GEO

**Figure 2:**
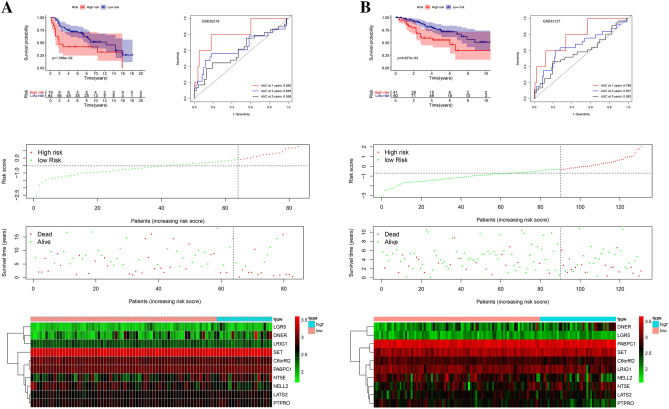
Validation of the signature in GEO. (**A**) Kaplan–Meier survival, ROC curve, and risk plot were used to verify the signature in the GSE30219. (**B**) Kaplan–Meier survival, ROC curve, and risk plot were used to validate the signature in the GSE42127.

To further verify the feasibility of the gene signature, we verified through the GEO database. In GSE20319 and GSE42127, the relationship between survival status, survival time, and the expression of the stem cell-related genes and risk score was consistent with the conclusion in TCGA. In GSE30219, the cut-offs value of the risk score between the high and low-risk group was − 0.085, the area under the ROC curve was 0.826, 0.638, and 0.599 in 1, 3, and 5-year survival rates, respectively. One of the more worthwhile was that in GSE42127, the cut-offs value of the risk score between the high and low-risk group was -0.316, the area under the ROC curve was 0.788, 0.657, and 0.582. Besides, The survival analysis in GSE30219 and GSE42127 revealed that the overall survival rate of the low-risk group was significantly better than that of the high-risk group (p < 0.05). This series of external verification fully demonstrated the feasibility and accuracy of our signature^18^ (Fig. 2).

### Subgroup analysis

**Figure 3:**
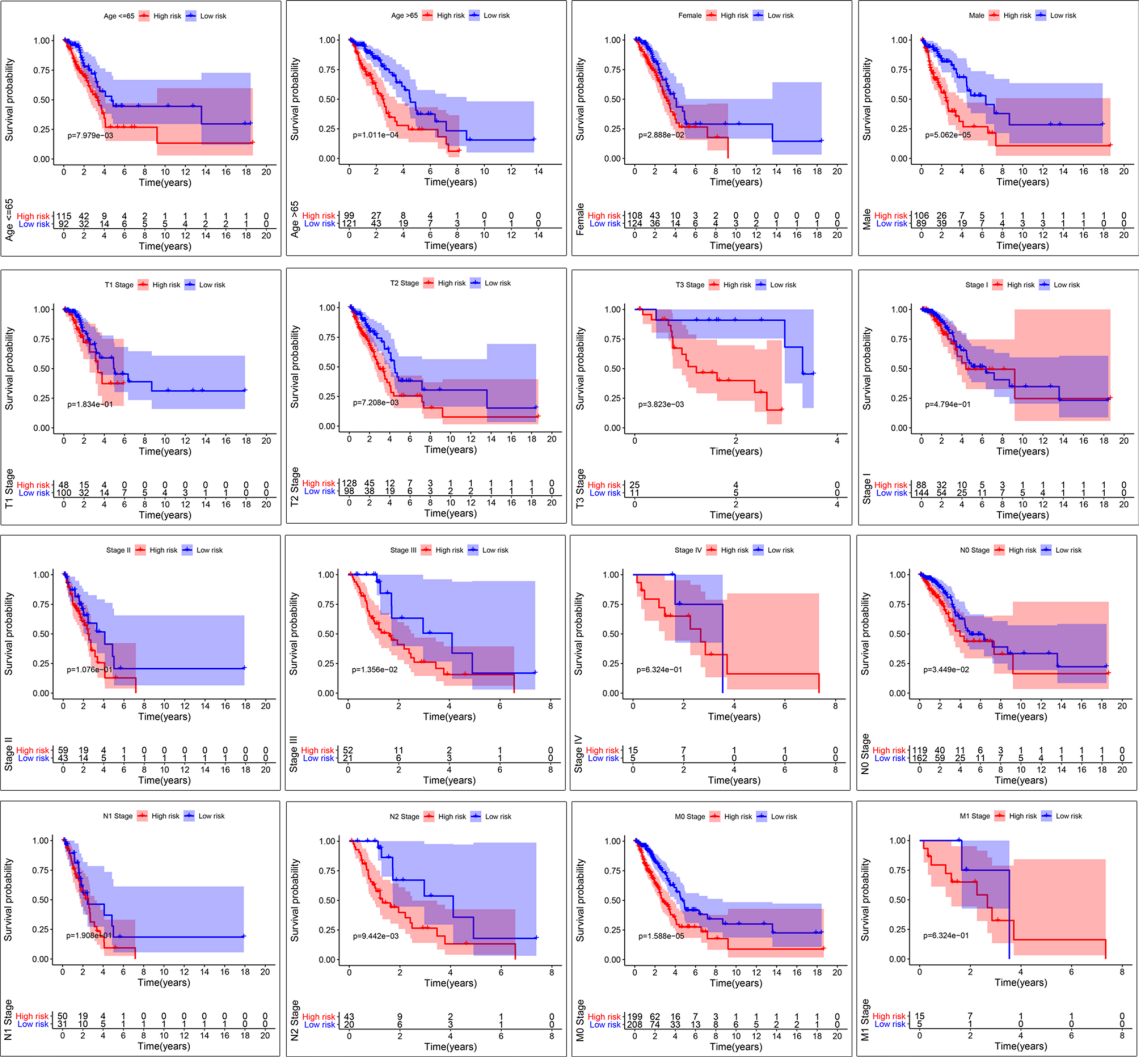
Subgroup analysis. Kaplan–Meier survival illustrated the overall survival of subgroups, which was stratified by age ≤ 65, age > 65, gender, and TNM stage.

**Figure 4:**
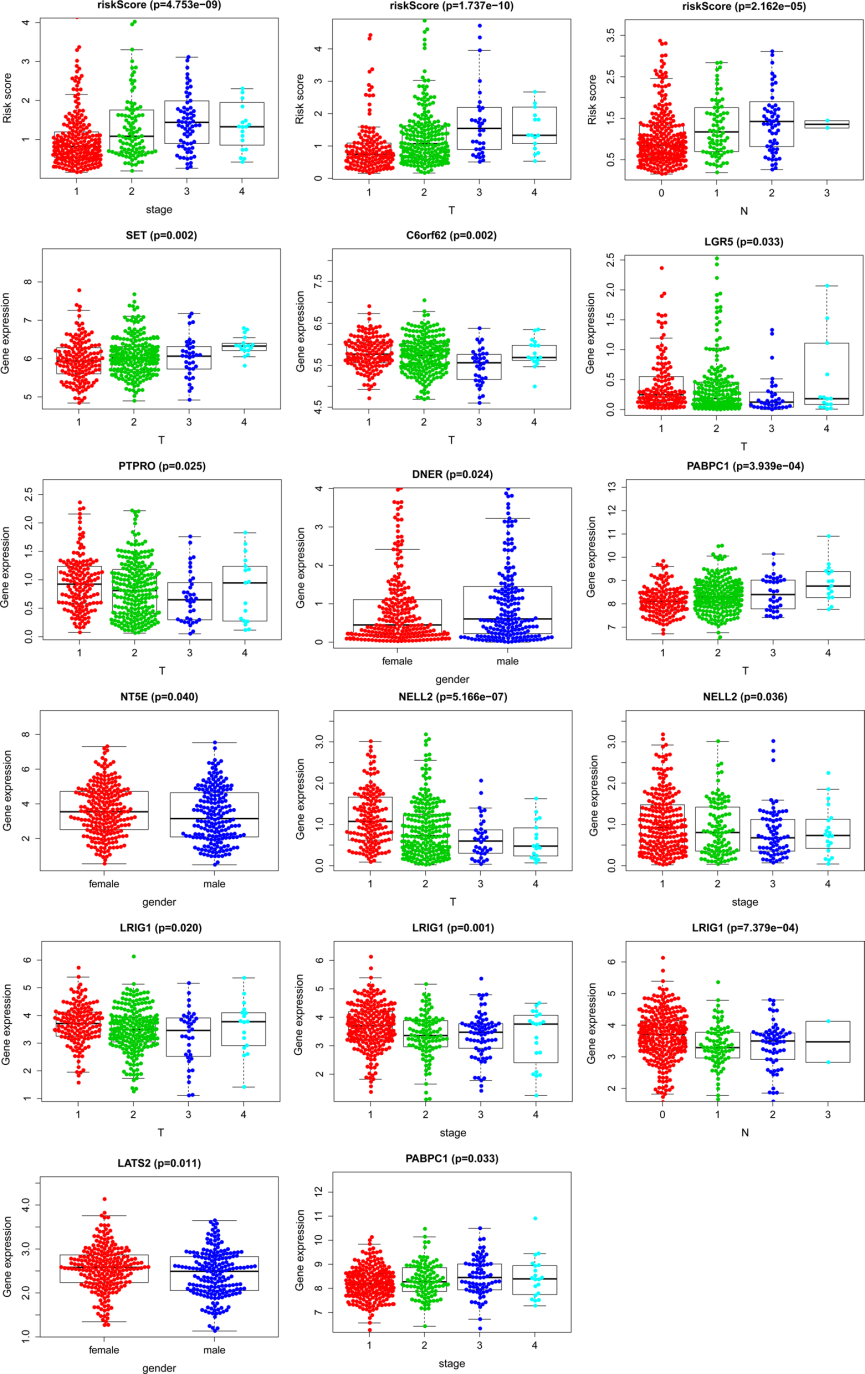
Subgroup analysis. The box plot showed the relationship among stem cell-related genes in the signature and each clinical subgroup.

We conducted a subgroup analysis to clarify the link between subgroups and risk grouping. The clinicopathologic features of LUAD patients in TCGA datasets were shown in Table 3. A further conclusion was drawn that all subgroups except N3 could identify high-risk and low-risk groups. In N3, there were only two patients, both of which belonged to the high-risk group. And in most subgroups, high- and low-risk groups had significant differences, such as age <  = 65, age > 65, female, male, stage III, T2, T3, N0, N2, and M0 (p < 0.05) (Fig. 3). P-value less than 0.05 was our criterion to judge whether it was meaningful^19^ (Fig. 4).

**Table 3:** Clinical information.

Clinical features	Category	Number(n = 427)	No. (%)
**Age**			
	< = 65	207	48.48%
	> 65	220	51.52%
**Gender**			
	Female	232	54.33%
	Male	195	45.67%
**Stage**			
	Stage I	232	54.33%
	Stage II	102	23.88%
	Stage III	73	17.10%
	Stage IV	20	4.69%
**T Stage**			
	T1	148	34.66%
	T2	226	52.93%
	T3	36	8.43%
	T4	17	3.98%
**N Stage**			
	N0	281	65.81%
	N1	81	18.97%
	N2	63	14.75%
	N3	2	0.47%
**M Stage**			
	M0	407	95.31%
	M1	20	4.69%
**Survival status**			
	Dead	147	34.43%
	Alive	280	65.57%

### Gene set enrichment analysis

**Figure 5:**
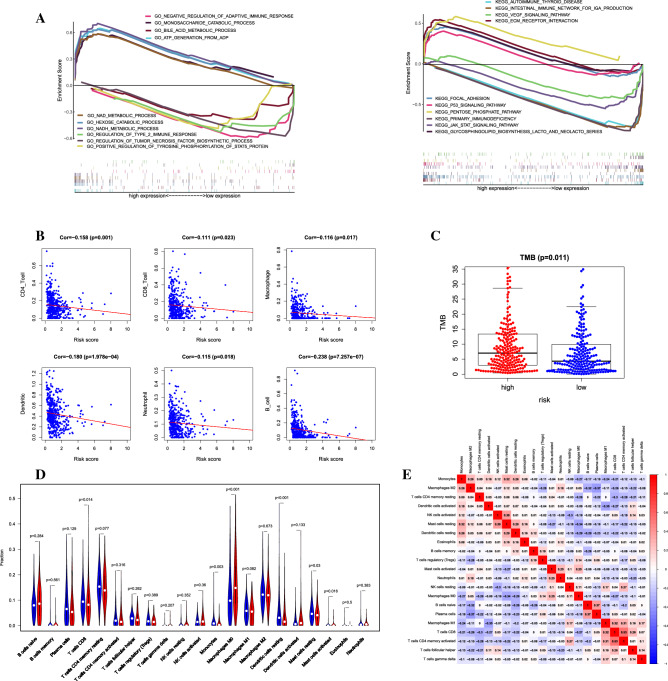
Gene Set Enrichment Analysis and Immune infiltration analysis. (**A**) GO term and KEGG pathway showed five positive correlation groups and five negative correlation groups, respectively. (**B**) TIMER indicated the correlations among the six immune cells and signature. (**C**) Difference analysis of TMB in high-risk and low-risk groups. (**D**) Composition of 21 kinds of immune cells in high-risk and low-risk groups. (**E**) Correlation heat map of 21 immune cells in LUAD.

The biological characteristics of the signature were confirmed by the analysis of the GO term and KEGG pathway. In GO term annotation, five categories were positively associated with the low-risk group, which were hexose catabolic process, NADH metabolic process, monosaccharide catabolic process, ATP generation from ADP, and NAD metabolic process. At the same time, five categories were negatively related to the low-risk group, which were negative regulation of adaptive immune response, regulation of tumor necrosis factor biosynthetic process, bile acid metabolic process, positive regulation of tyrosine phosphorylation of STAT5 protein, and regulation of type 2 immune response. In the KEGG pathways, five pathways were positively associated with the low-risk group, such as ECM receptor interaction, focal adhesion, glycosphingogolipid biosynthesis latco, and neolatco series, pentose phosphate pathway, and p53 signaling pathway. While five pathways were negatively related to the low-risk group, like JAK start signaling pathway, primary immunodeficiency, VEGF signaling pathway, and intestinal immune network for IGA production^20^ (Fig. 5A).

### Immune infiltration analysis

TIMER database, which provides six types of immune cell infiltration, uses RNA-Seq expression profiling data to detect immune cell infiltration in tumor tissue. The signature showed a negative correlation with the levels of B cells, CD4 T cells, CD8 T cells, Dendritic cells, Macrophages, and neutrophil cells (p < 0.05) (Fig. 5B). Tumor mutation burden (TMB) is defined as the total number of somatic gene coding errors, gene insertion, or deletion errors detected per million bases. TMB was obtained according to the above calculation method based on the varscan.maf file provided in the TCGA database. According to the calculated mutation burden value, we found that it had significant differences in the low-risk and high-risk groups (Fig. 5C). These situations revealed that our signature was indeed related to immune cells. In addition, we characterize the cellular composition of the tumor-infiltrating immune cells through the CIBERSORT method. Compared with the high-risk group, CD8 T cells, monocytes, resting dendritic cells, and resting mast cells had higher expressions (p< 0.05), while M0 macrophage had lower expression (p< 0.001) (Fig. 5D). CD4 memory activated T cells and CD8 T cells had the highest positive correlation (R = 0.53), which implied that there was a mutual effect between them. While plasma and M2 Macrophages had the highest negative correlation (R = − 0.37) that suggested they were antagonistic to each other^21^ (Fig. 5E).

### Therapeutic efficacy analysis

**Figure 6:**
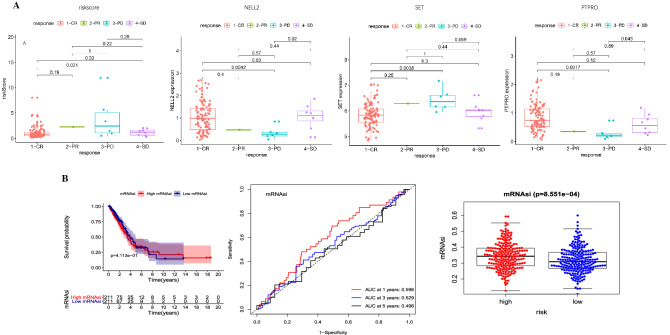
Analysis of therapeutic efficacy and correlation analysis of stem cell index. (**A**) Box plot suggests the links between the p-value of the difference between any two groups. (**B**) Kaplan–Meier survival, ROC curve, and box plot were used to demonstrate the risk prediction of signature-based on the stem cell index.

Some patients in the TCGA database recorded the results of the first assessment of the efficacy of radiotherapy and chemotherapy. This part of the result was obtained from the cbioportal database. Among them, 126 patients recorded the results of the first treatment after radiotherapy and chemotherapy. At the same time, we tracked the evaluation of efficacy, 111 cases were complete response (CR), only one case was the partial response (PR), eight cases were stable disease (SD), and seven cases were progressive disease (PD). The three genes of the signature had significant differences in the efficacy of the different drug (p < 0.05). We integrated this aspect into our research to evaluate our signature from multiple perspectives. By calculating the relationship between the signature and the efficacy of radiotherapy and chemotherapy, the predictive power of the signature could be calculated^22,23^ (Fig. 6A).

### Relationship between signature and mRNAsi

There were already clear articles that calculated the mRNAsi of 1174 genes. We matched the known mRNAsi with the samples and divided our patients into two groups by the median value of mRNAsi (high-mRNAsi group and low-mRNAsi group)^24^. It was found that mRNAsi could not effectively distinguish high- and low-mRNAsi in LUAD and the area under the ROC curve still had a certain gap compared with our signature. However, the mRNAsi of the high-risk group in our signature was also significantly higher than that of the low-risk group (P < 0.01). This also verified that our signature was stem cell characteristic^25,26^ (Fig. 6B).

### Clinical correlation analysis

**Figure 7:**
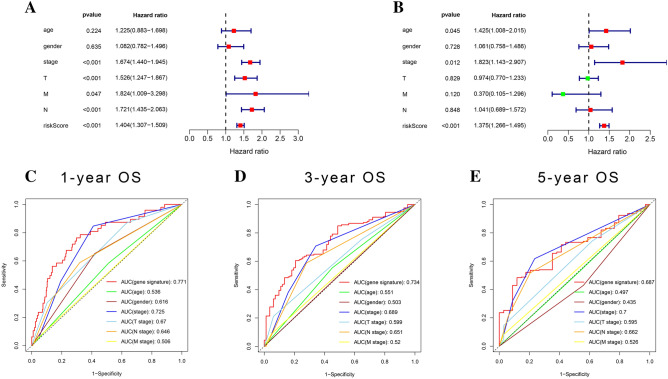
Clinical relevance. (**A**,**B**) Univariate and Multivariate Cox regression analysis of clinical factors related to overall survival in the TCGA cohort. (**C**–**E**) ROC curve demonstrated the risk prediction compared with other clinical factors in the TCGA cohort.

**Figure 8:**
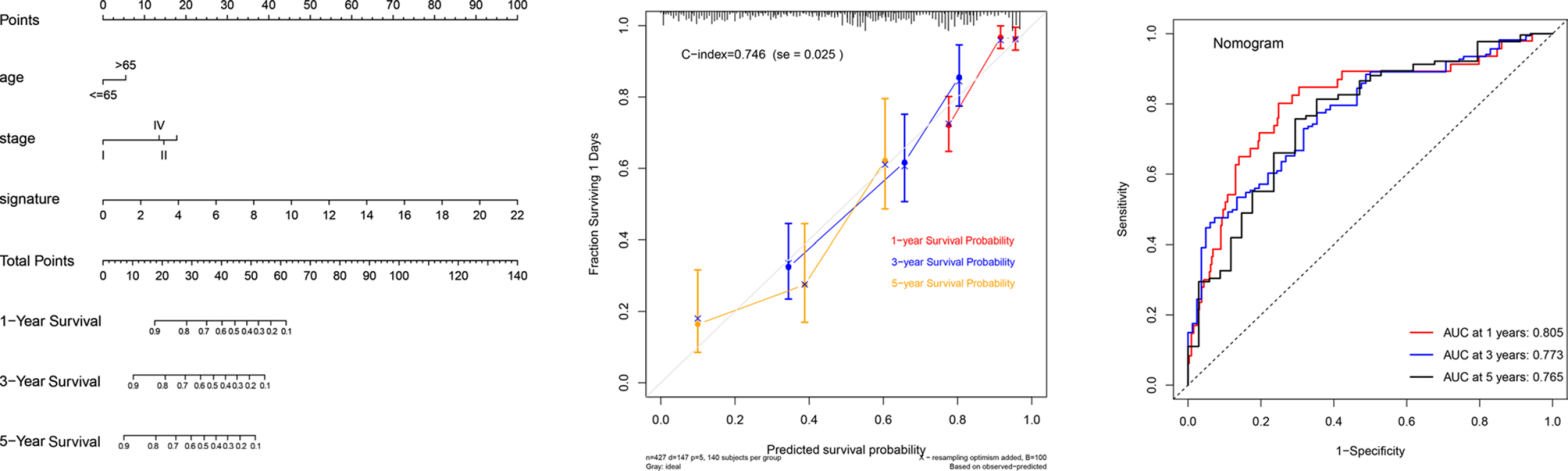
Construction of nomogram. The nomogram contained age, stage, signature containing ten stem cell-related genes. The x-axis of the calibration chart was the predicted recurrence probability result, and the y-axis was the actual recurrence probability. ROC analysis detected the accuracy of prediction and inspection.

**Figure 9:**
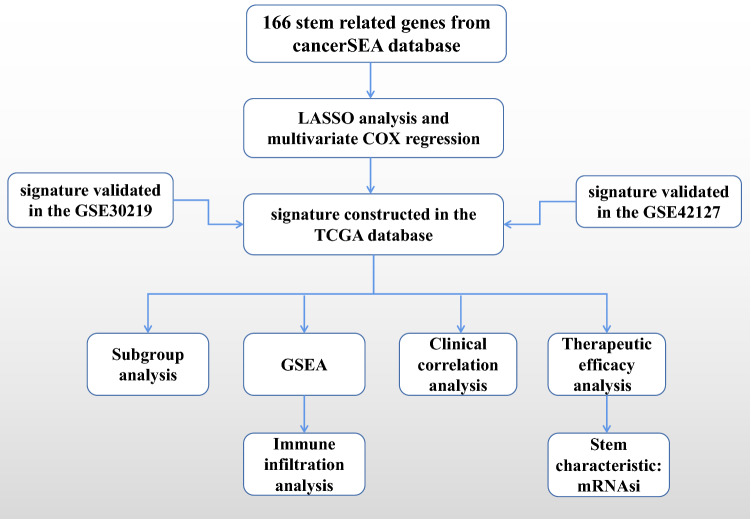
Flow chart. The flow chart was drawn to show the thought and process of our research.

The univariate Cox regression showed factors related to prognosis like a stage, T, M, N, and risk score (p < 0.05), while multivariate Cox regression showed that only stage and risk score were significant independent risk factors of LUAD. Compared with other clinical factors, the area under the ROC curve of the signature in each period was the largest, which implied that compared with other clinical factors, the predictive ability of the gene signature we constructed was optimal (Fig. 7A,B). The areas under the ROC curve for 1-year, 3-year, and 5-year OS were 0.771, 0.734, and 0.687, which implied that our signature had excellent predictive power^27,28^ (Fig. 7C–E). We constructed a nomogram that could predict 1, 3 and 5-year OS by signature and other clinical factors. The 1, 3 and 5-year OS probability calibration curves showed that the OS predicted by nomogram was in good agreement with the actual OS of LUAD patients. The ROC curve in the nomogram showed that the 1, 3 and 5-year forecast values were 0.805, 0.773, and 0.765^29^ (Fig. 8). The workflow of our study was shown in Fig. 9, which was used to display our thought and process of our study.

## Discussion

**Figure 10:**
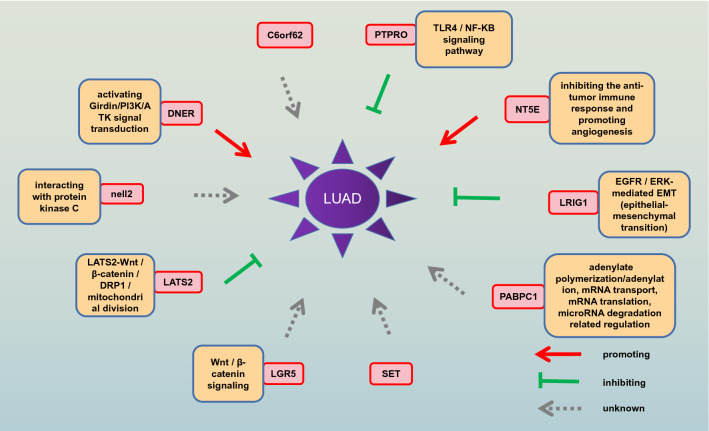
Schematic diagram. The schematic diagram was drawn to reflect the genes in the signature how to guide the progression of LUAD. The red arrow represented promoting the progression of LUAD, the green arrow represented inhibiting the progression of LUAD, and the gray arrow represented the impact on LUAD not yet.

Despite the dramatic progress in diagnosis and treatment, the prognosis of advanced lung adenocarcinoma is still unsatisfactory. With the development of clinical management of lung cancer, some prognostic factors are well characterized, such as age, grade, and TNM grade. Cancer stem cells refer to cells that have self-renewal capacity and can produce heterogeneous tumor cells, which play a significant role in tumor survival, proliferation, metastasis, and recurrence. Cancer stem cells or tumor-initiating cells are considered to be the main drivers of disease progression and treatment resistance across various cancer types. Therefore, stem cell-related genes that were used to construct our signature also had these characteristics. This was why we considered using these genes to build a signature to facilitate the prediction and precise treatment of lung cancer. The research on the mechanism of stem cell-related genes has been pervasive, but there is no experiment to build these stem cell-related genes into a signature. DNER is a neuron-specific transmembrane protein with extracellular EGF-like repeat sequences, which promotes the metastasis and proliferation of cancer cells by activating Girdin/PI3K/ATK signal transduction^30–32^. NELL2s is a rich glycoprotein that contains EGF-like domains in nerve tissues, which interact with protein kinase C and has multiple physiological functions. Hypermethylation of promoter silences NELL2 and affects the progression of renal cell carcinoma^33–35^. LATS2, as a potential tumor suppressor, is a significant mediator of the apoptosis response pathway. LATS2-Wnt/β-catenin/DRP1/mitochondrial division is identified as a signaling pathway that promotes cancer cell death^36,37^. LGR5 is a promising marker of intestinal stem cells and cancer stem cells. Intestinal stem cell marker LGR5 is a receptor for R-spongin, and its role is to enhance Wnt signaling in hyperplastic crypts. Wnt pathway plays a significant key in ISC self-renewal by inducing RSPO receptor LGR5 expression. An abnormal increase in LGR5 expression may represent one of the most common molecular changes in some human cancers, resulting in long-term enhancement of canonical Wnt/β-catenin signaling^38–40^. PTPRO is a tumor suppressor and is abnormally expressed in various malignant tumors. PTPRO causes ulcerative colitis through TLR4/NF-KB signaling pathway and plays a role in liver fibrosis by affecting PDGF signaling in HSC activation. It is noteworthy that PTPRO is a new candidate gene for emphysema with severe obstruction^41,42^. LRIG1, a transmembrane protein, has a tumor-suppressive effect, and its expression is down-regulated in a variety of cancers. It can antagonize epidermal growth factor receptor signaling in epithelial tissues and inhibit cell invasion, migration, VM (angiogenesis simulation) by regulating EGFR / ERK-mediated EMT (epithelial-mesenchymal transition)^43,44^. PABPC1 can combine with adenylate-rich sequences in mRNA under the action of high affinity, which plays an important role in post-transcriptional regulation of genes and is also involved in many metabolic pathways of mRNA, including adenylate polymerization/adenylation, mRNA transport, mRNA translation, microRNA degradation related regulation^45^. NT5E is a ubiquitously expressed glycosylphatidylinositol-fixed glycoprotein, which can convert extracellular adenosine 5′-monophosphate to adenosine, and promote tumor development by inhibiting the anti-tumor immune response and promoting angiogenesis^46,47^. A schematic gram was used to display that the genes in the signature how to guide the progression of LUAD (Fig. 10). As far as AUC is concerned, the signature-based on ten stem cell-related genes is indeed a strong complement to TNM staging, but this conclusion needs further verification with multi-center and larger samples.

GSEA proved that the constructed signature did involve related cancer pathways. P53 is a tumor suppressor protein that regulates the expression of various genes, including apoptosis, growth inhibition, differentiation, inhibition of cell cycle progression, and accelerated DNA repair, genotoxicity, and senescence after cellular stress. Like all other tumor suppressors, the P53 gene normally slows or monitors cell division. The JAK/STAT signaling pathway is involved in numerous significant biological processes such as cell differentiation, proliferation, migration, apoptosis, survival, and immune regulation. Besides, the JAK/STAT signaling pathway also participates in the drug treatment of anemia, thrombocytopenia, neutropenia, and antiviral. With immune infiltration analysis, we found that the signature regulates the immunity of lung adenocarcinoma through CD4 T cell, which can interfere with the immune response of the immune system to the tumor, participate in the immune escape of the tumor, induce the immune tolerance of the tumor, and promote the occurrence and development of the tumor.

## Conclusion

In conclusion, the signature could effectively predict the efficacy of chemotherapy in patients with LUAD, and a nomogram based on signature might accurately predict the prognosis of patients with LUAD. The signature-based on stem cell-related genes might be contributed to predicting the prognosis of patients with LUAD. Further research should be devoted to the functional analysis of our research results and verification in clinical trials.

## Supplementary Information

Supplementary Information 1.

## Data Availability

All data were from TCGA and GEO, which are publicly available. Data and code available.

## Abbreviations

LUAD: Lung adenocarcinoma
LASSO: Least absolute shrinkage operator
ROC: Receiver operating characteristic
AUC: Area under the curve
TIIC: Tumor-infiltrating immune cell
TCGA: The cancer genome atlas
GEO: Gene expression omnibus
RECIST: Response evaluation criteria in solid tumors
CR: Complete response
PR: Partial response
SD: Stable disease
PD: Progressive disease
mRNAsi: MRNA stemness indice
OS: Overall survival
GO: Gene ontology
KEGG: Kyoto encyclopedia of genes and genomes
GSEA: Gene set enrichment analysis
