# Supplementary material for: Prognostic signature of lung adenocarcinoma based on stem cell-related genes
Source: Sci Rep. 2021 Jan 18;11:1687. doi: 10.1038/s41598-020-80453-4 (PMC7814011; doi:10.1038/s41598-020-80453-4)
Supplement: Supplementary file 1 — Supplementary Information 1. [file 41598_2020_80453_MOESM1_ESM.zip › a single composite supplementary information file/figure captions.docx]

**Figure captions**

Fig. 1 **Construction of signature.** (A) The risk curve in the TCGA cohort displays the patients’ risk score, survival time, and status and expression of stem cell-related genes. The scales represent the expression level of each gene in each sample, which is established based on z-score transformed expression data. (B) ROC curve illustrates the risk prediction of the signature for 1, 3, and 5 years in the TCGA cohort. (C) Kaplan-Meier survival reveals the overall survival among different risk stratification groups.

Fig. 2 **Validation of the signature in GEO.** (A) Kaplan-Meier survival, ROC curve, and risk plot were used to verify the signature in the GSE30219. (B) Kaplan-Meier survival, ROC curve, and risk plot were used to validate the signature in the GSE42127.

Fig. 3 **Subgroup analysis.** Kaplan-Meier survival illustrates the overall survival of subgroups, which was stratified by age ≤ 65, age > 65, gender, and TNM stage.

Fig. 4 **Subgroup analysis.** The box plot shows the relationship between stem cell-related genes in the signature and each clinical subgroup.

Fig. 5 **Gene Set Enrichment Analysis** **and Immune infiltration analysis.** (A) GO term and KEGG pathway show five positive correlation groups and five negative correlation groups, respectively. (B) TIMER indicates the correlations between the six immune cells and signature. (C) Difference analysis of TMB in high-risk and low-risk groups. (D) Composition of 21 kinds of immune cells in high risk and low-risk groups. (E) Correlation heat map of 21 immune cells in LUAD.

Fig. 6 **Relationship between the signature and therapeutic efficacy and stem cell index.** (A) Box plot suggests the links between the p-value of the difference between any two groups. (B) Kaplan-Meier survival, ROC curve, and box plot were used to demonstrate the risk prediction of signature-based on the stem cell index.

Fig. 7 **Clinical relevance.** (A-B) Univariate and Multivariate Cox regression analysis of clinical factors related to overall survival in the TCGA cohort. (C-E) ROC curve demonstrates the risk prediction compared with other clinical factors in the TCGA cohort.

Fig. 8 **Construction of nomogram.** The nomogram contains age, stage, signature containing ten stem cell-related genes. The x-axis of the calibration chart is the predicted recurrence probability result, and the y-axis is the actual recurrence probability. ROC analysis detects the accuracy of prediction and inspection.

Fig. 9 **Flow chart.** The flow chart was drawn to show the thought and process of our research.

Fig. 10 **Schematic diagram.** The schematic diagram was drawn to reflect the genes in the signature how to guide the progression of LUAD. The red arrow represented promoting the progression of lung adenocarcinoma, the green arrow represented inhibiting the progression of lung adenocarcinoma, and the gray arrow represented the impact on lung adenocarcinoma not yet.

Supplementary figure **Lasso regression analysis.** Lasso method for feature selection.
