# Supplementary material for: Prognostic signature of lung adenocarcinoma based on stem cell-related genes
Source: Sci Rep. 2021 Jan 18;11:1687. doi: 10.1038/s41598-020-80453-4 (PMC7814011; doi:10.1038/s41598-020-80453-4)
Supplement: Supplementary file 1 — Supplementary Information 1. [file 41598_2020_80453_MOESM1_ESM.zip › a single composite supplementary information file/title page.docx]

**Prognostic signature of lung adenocarcinoma based on stem cell-related genes**

Subtitle: Prognostic signature based on stem cell-related genes

Zhanghao Huang^1,2†^, Muqi Shi^4†^, Hao Zhou^1,2^, Jinjie Wang^1,2^, Hai-Jian Zhang^3*^, Jia-Hai Shi^1,2*^

^1^ Nantong Key Laboratory of Translational Medicine in Cardiothoracic Diseases, and Research Institution of Translational Medicine in Cardiothoracic Diseases, Affiliated Hospital of Nantong University, Nantong 226001, Jiangsu, China;

^2^ Department of Thoracic Surgery, Affiliated Hospital of Nantong University, Nantong 226001, Jiangsu, China;

^3^ Research Center of Clinical Medicine, Affiliated Hospital of Nantong University, Nantong 226001, Jiangsu, China;

^4^ Medical College of Nantong University, Nantong 226001, Jiangsu, China;

*Correspondence: Hai-Jian Zhang, hjzhang@ntu.edu.cn; Jia-Hai Shi, [sjh@ntu.edu.cn](mailto:sjh@ntu.edu.cn);

^†^These authors contributed equally to this work.

Tel.: 86-0513-85052331

Fax: +86-0513-85519820;

mail: [1931320141@stmail.ntu.edu.cn](mailto:1931320141@stmail.ntu.edu.cn)
