# Supplementary figures and images for: Prognostic signature of lung adenocarcinoma based on stem cell-related genes
Source: Sci Rep. 2021 Jan 18;11:1687. doi: 10.1038/s41598-020-80453-4 (PMC7814011; doi:10.1038/s41598-020-80453-4)

Figure 1


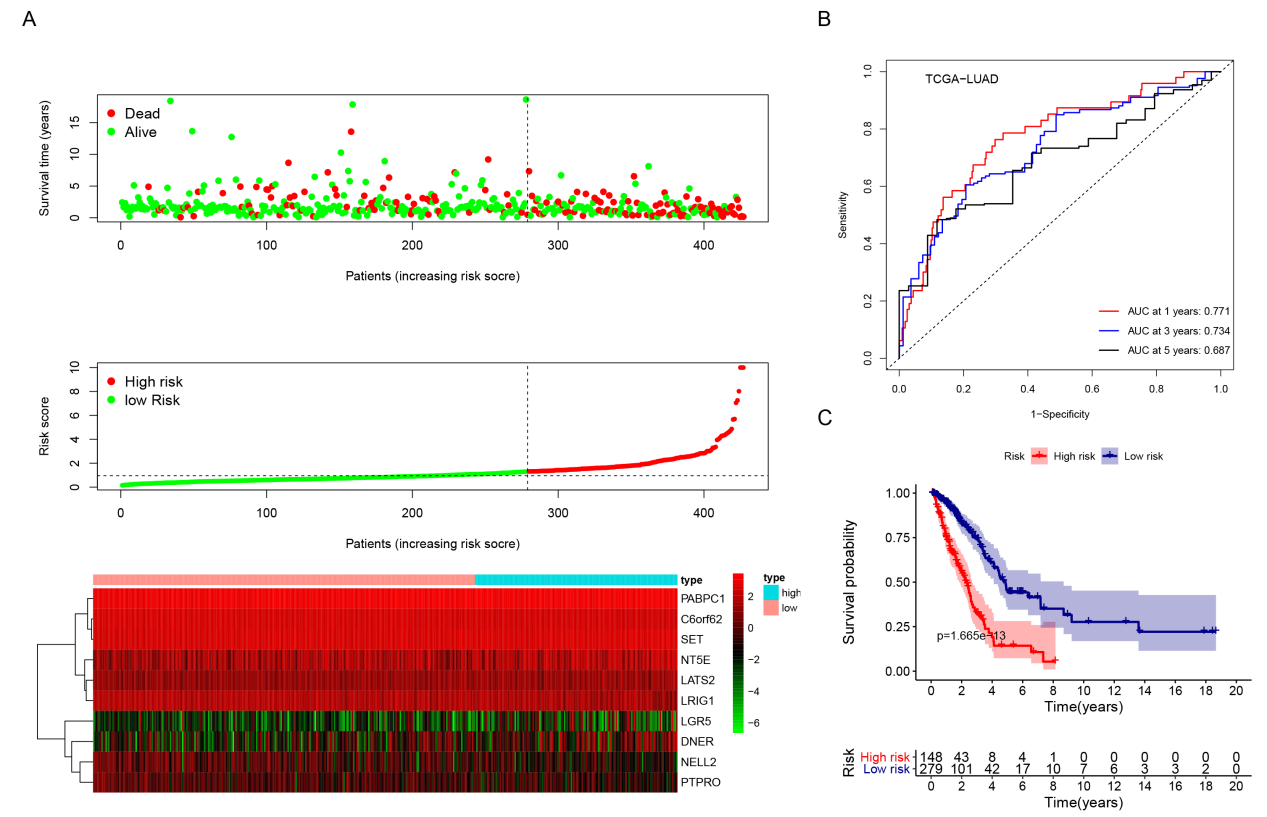


Figure 2


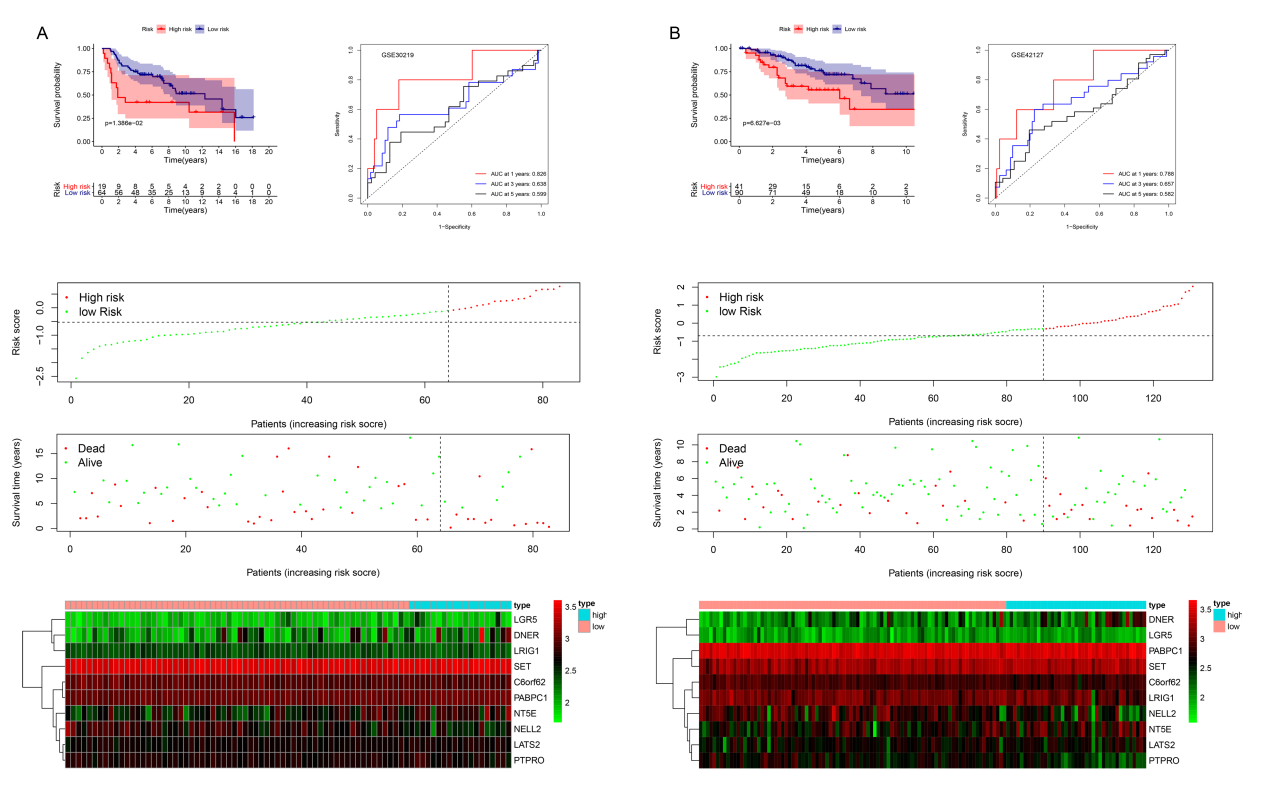


Figure 3


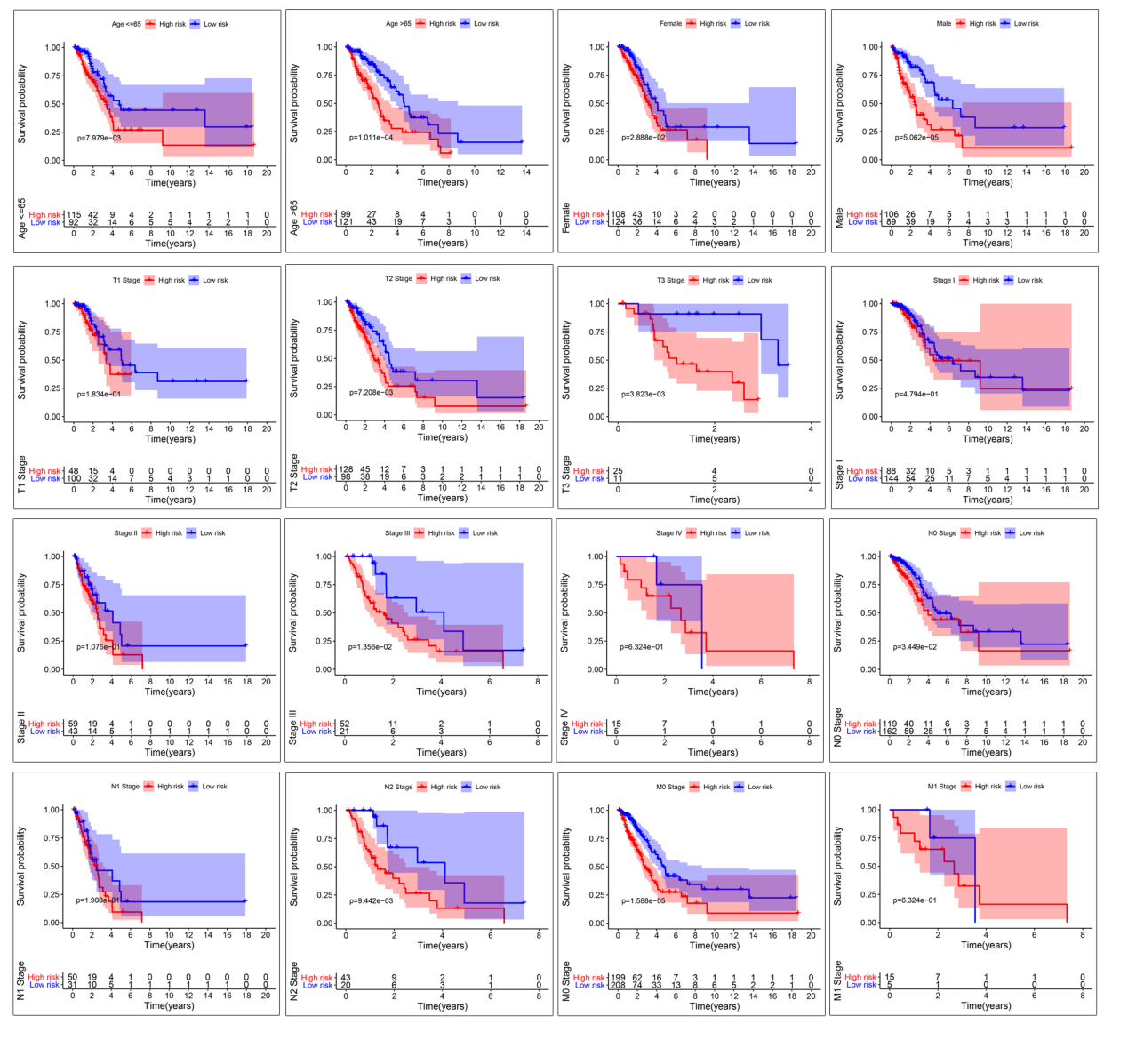


Figure 4


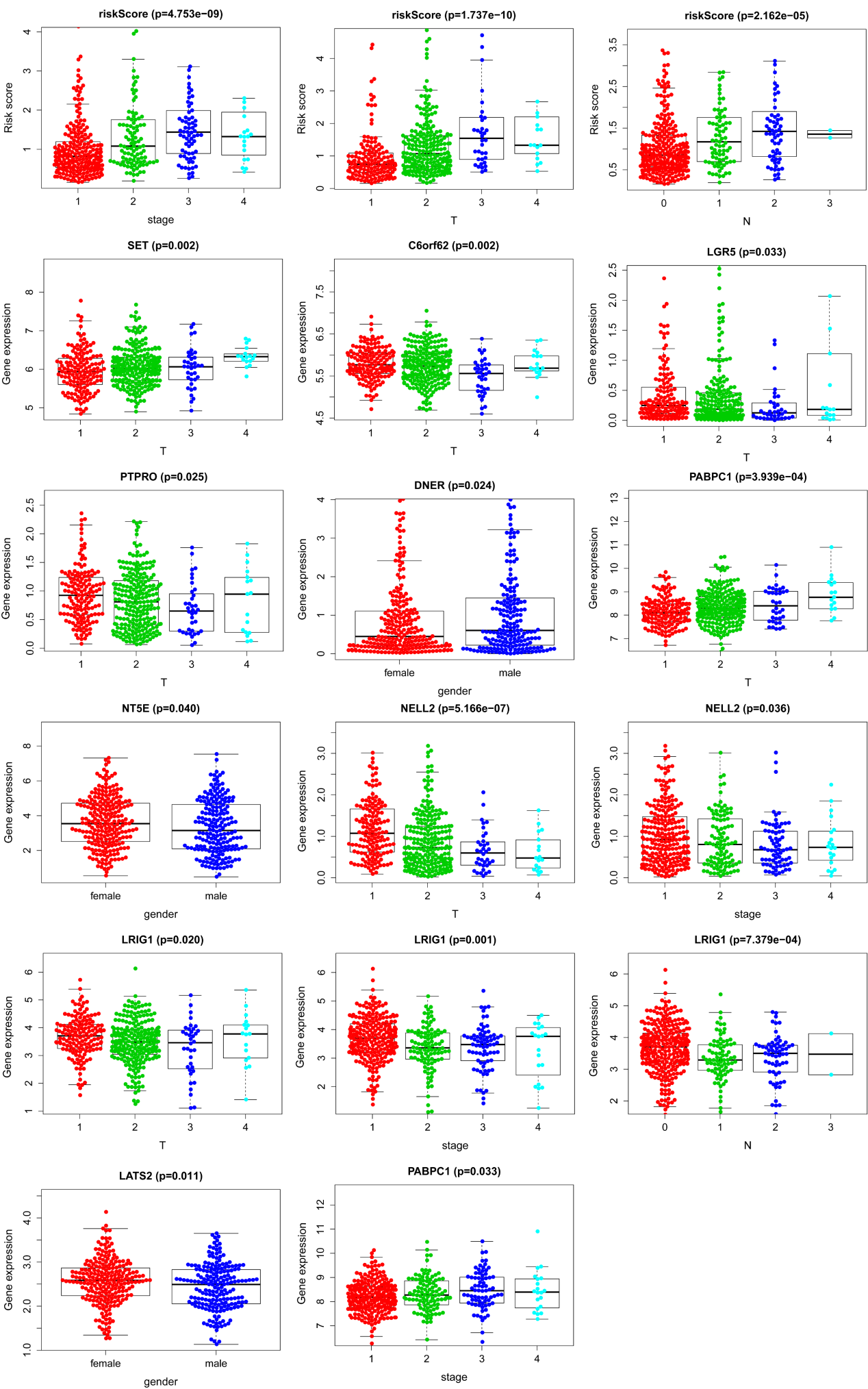


Figure 5


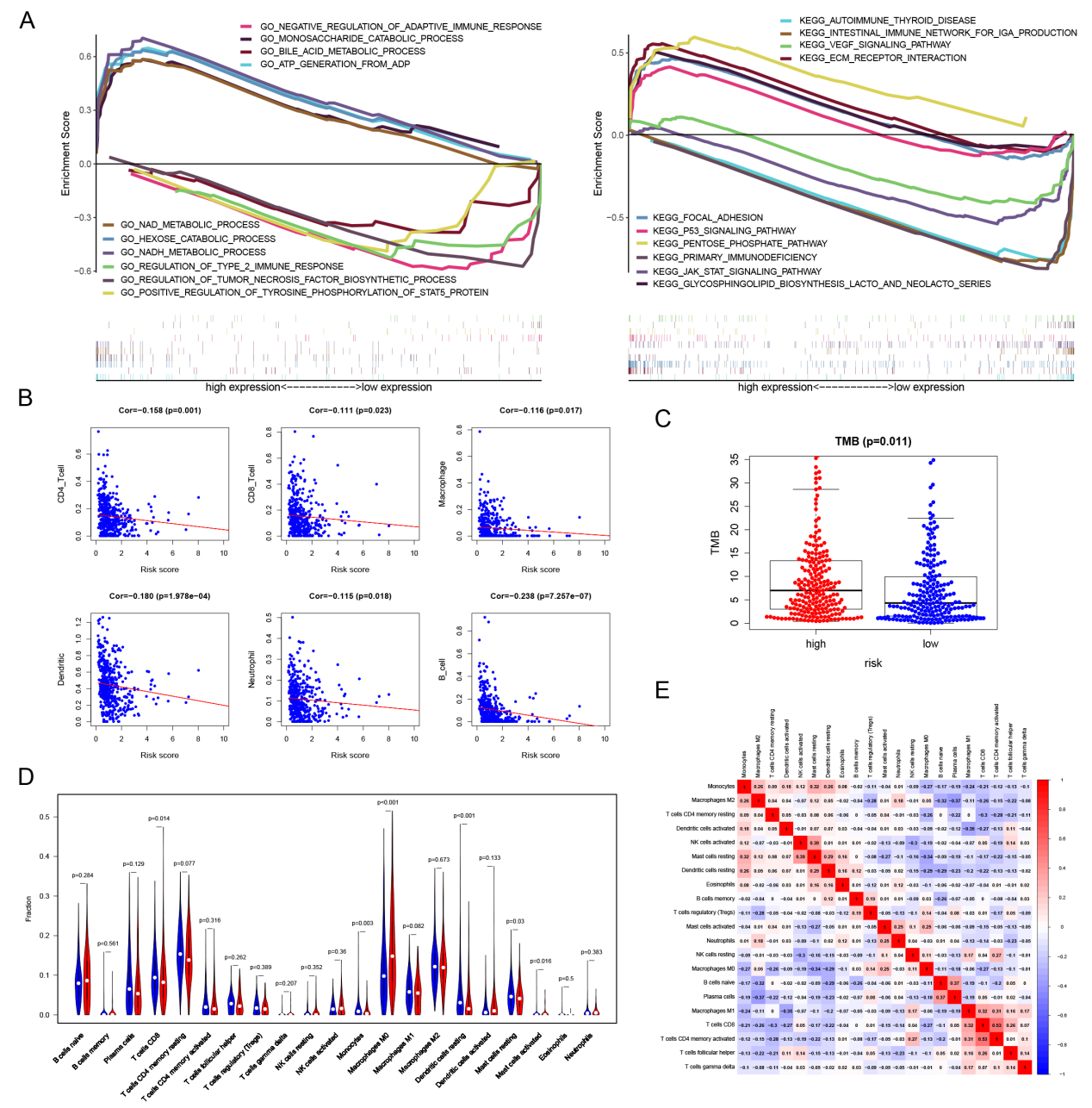


Figure 6


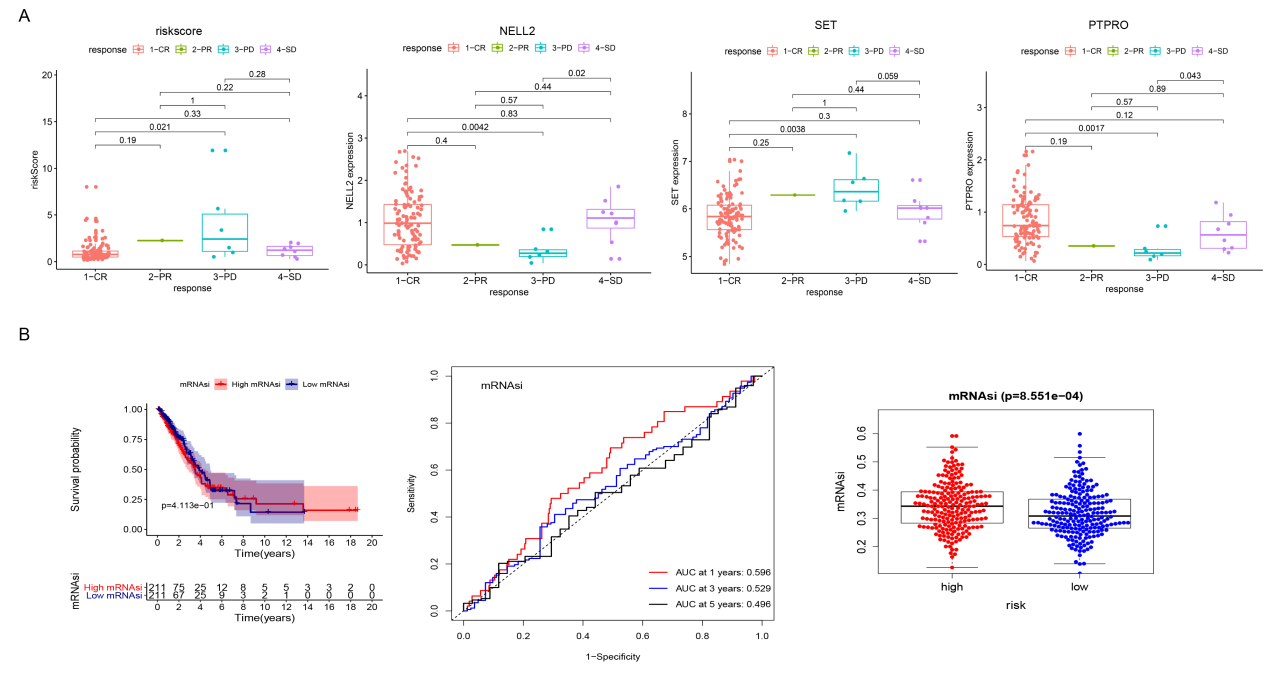


Figure 7


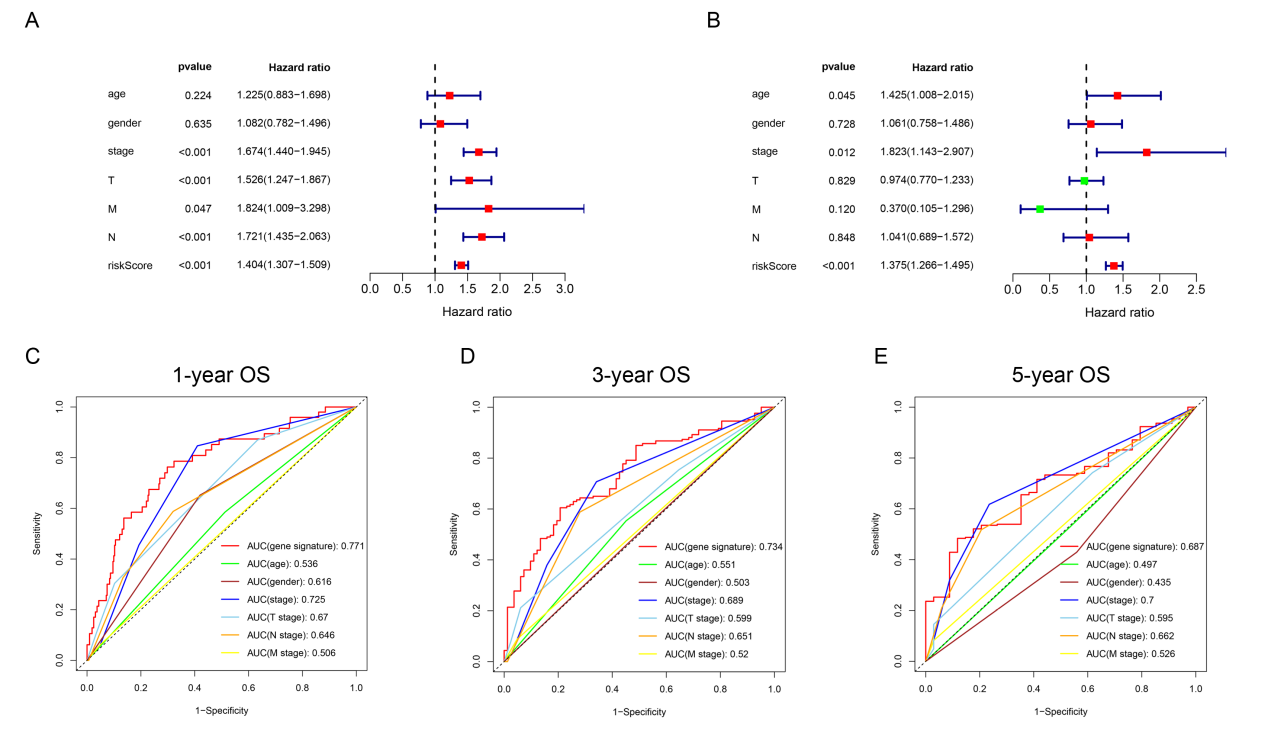


Figure 8


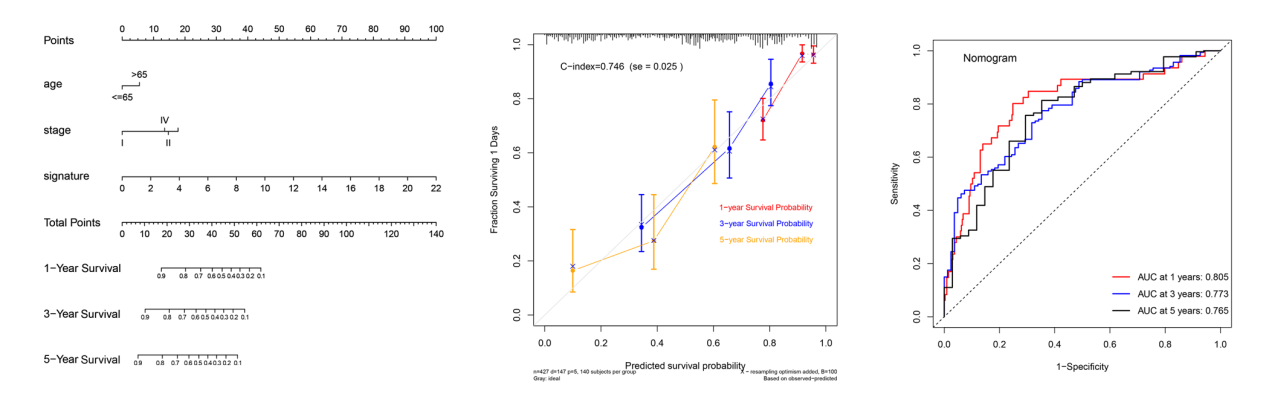


Figure 9


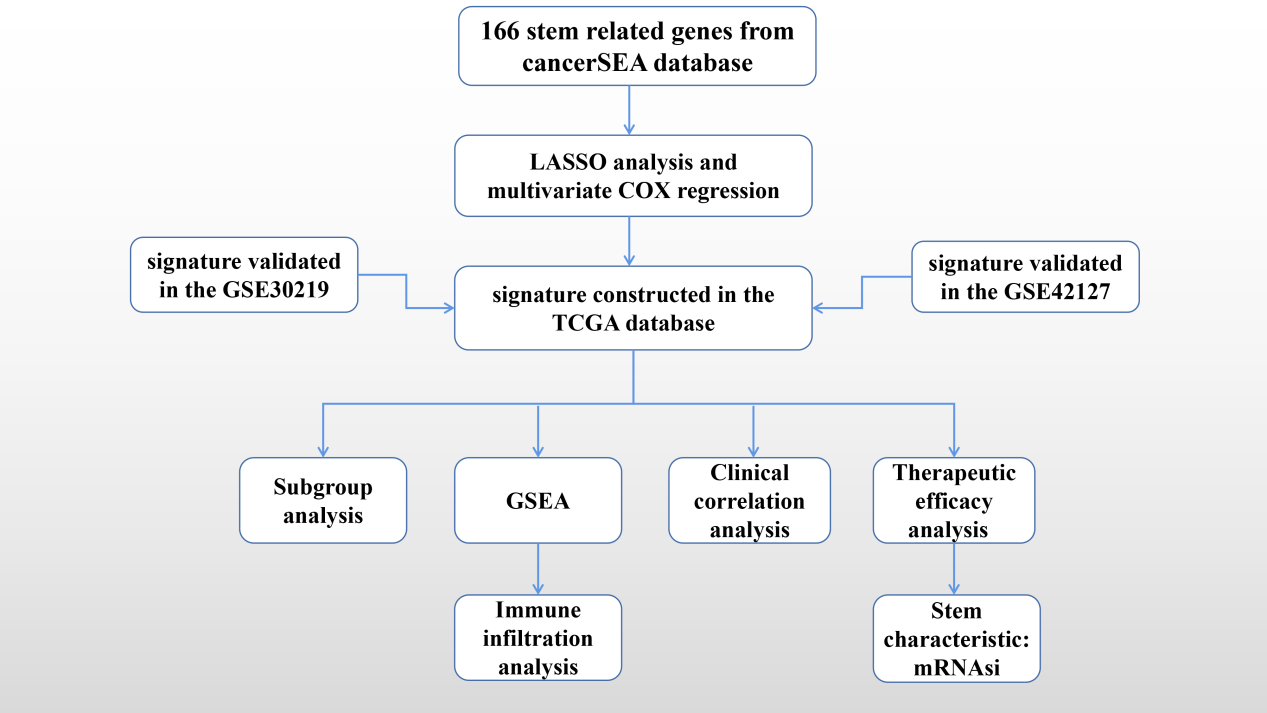


Figure 10


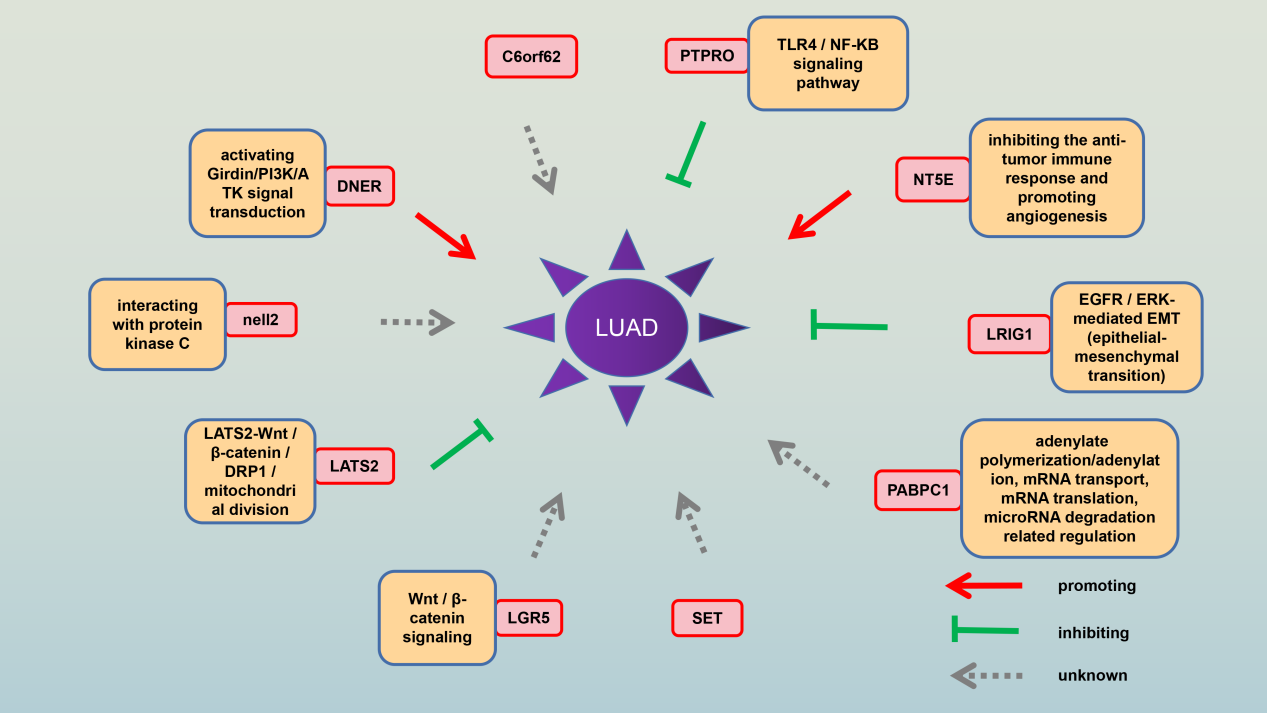


Supplementary figure


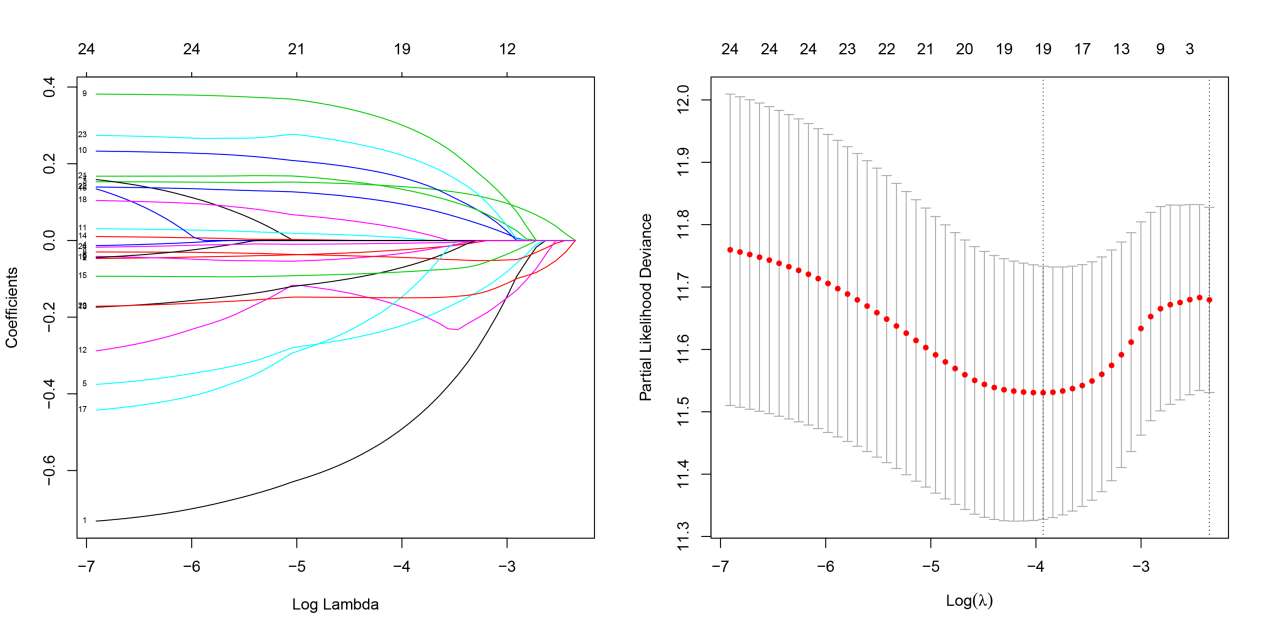

Supplement: Supplementary file 1 — Supplementary Information 1. [file 41598_2020_80453_MOESM1_ESM.zip › a single composite supplementary information file/figure images.docx]
